# Susceptibility and resilience to cyber threat: Findings from a scenario decision program to measure secure and insecure computing behavior

**DOI:** 10.1371/journal.pone.0207408

**Published:** 2018-12-12

**Authors:** Carl F. Weems, Irfan Ahmed, Golden G. Richard, Justin D. Russell, Erin L. Neill

**Affiliations:** 1 Department of Human Development and Family Studies, Iowa State University, Ames, Iowa, United States of America; 2 Department of Computer Science, Virginia Commonwealth University, Richmond, Virginia, United States of America; 3 Division of Computer Science and Engineering, Louisiana State University, Baton Rouge, Louisiana, United States of America; 4 Department of Psychology, Iowa State University, Ames, Iowa, United States of America; Victoria University, AUSTRALIA

## Abstract

Interest in the individual differences underlying end user computer security behavior has led to the development of a multidisciplinary field of research known as behavioral information security. An important gap in knowledge and the motivation for this research is the development of ways to measure secure and insecure cyber behavior for research and eventually practice. Here we report a study designed to develop a technique for assessing secure and insecure cyber behavior for broad research use. The Susceptibility and Resilience to Cyber Threat (SRCT) is an immersive scenario decision program. The SRCT measures susceptibility to cyber threat and malicious behavior as well protective resilience actions via participant responses/decisions to emails, interactions with security dialogs, and computer actions in a real-world simulation. Data were collected from a sample of 190 adults (76.3% female), between the ages of 18–61 (mean age = 26.12). Personality, behavioral tendencies, and cognitive preferences were measured with standard previously validated protocols and self-report measures. Factor analysis suggested a 5 item secure actions scale and a 9 item insecure actions scale as viable to extract from the SRCT responses. Statistically analyzable distributions of secure and insecure cyber behaviors were obtained, and these subscales demonstrated acceptable internal consistency as hypothesized. Associations between SRCT scales and other indices of cyber behavior, as well as self-reported personality, were lower than predicted, suggesting that past research reporting links between self-reports of personality and self-reported cyber-behavior may be overestimating the links for actual cyber actions. However, our exploratory analyses suggest discrepancies between self-report and actions in the SRCT may be an interesting avenue to explore. Overall, results were consistent with theorizing and suggest the technique is viable as a construct measure in future research or as an outcome variable in experimental intervention designs.

## Introduction

The need for multifaceted cybersecurity efforts in academic, government, and business communities have resulted in calls for greater understanding of personality, behavioral, and cognitive factors in secure computing behavior [1–4]. Interest in the individual differences underlying end user computer security behavior has led to the development of a multidisciplinary field of research known as behavioral information security (BIS). BIS integrates psychology, behavioral science, and computer science to explore links between human traits and cybersecurity [5,6]. One premise of this research is that there are particular behavioral characteristics, cognitive preferences, and personality factors that may be associated with secure versus insecure cyber behavior [7,8]. An important avenue of investigation in this field—and the motivation for this research—is the development of ways to measure secure and insecure cyber behavior for research and eventually practice.

While personal computer and network protection systems can defend against external cyber threats, most organizations still depend on the end user to engage in secure computing practices to minimize vulnerability [9]. Internal threats to network/organization security can occur when a user *passively* (or inadvertently) fails to abide by organizational (or best practice) security procedures or *actively* attempts to thwart security (i.e., insider attack). Such internal threats are theoretically related to behavior, cognitive styles, or personality traits of system users. Development of a conceptual definition of cyber secure/insecure behavior of system users is a first step in designing the measurement of constructs in psychological science (i.e., defining what should be measured). Secure/insecure computer behavior falls under the overarching concept of cybersecurity. While scholars continue to debate the criteria and behaviors necessary to define a “cybersecure” state [10], the United States Committee on National Security Systems (CNSS) provides the following simple definition on which we chose to base construct development: “The ability to protect or defend the use of cyberspace from cyber-attacks,” [11]. An individual’s secure or insecure cyber behavior would therefore constitute actions that promote or disrupt this ability to protect or defend.

Behavioral science researchers commonly measure constructs using observations, self-report, or indirect methods (i.e., the “how to” measure question). In observational studies one records the actual actions or behaviors in question (e.g., did the individual delete or open a phishing email). For example, Halevi et al. [12], measured personality traits (i.e., Big Five personality traits of openness, conscientiousness, extraversion, agreeableness, and neuroticism) in a sample of participants who were sent a phishing e-mail by the experimenters. The authors reported a correlation between neuroticism (tendency to be anxious, depressed, etc.) and falling victim to the attack (i.e., participants high in neuroticism were more likely to be successfully phished). Chen, YeckehZaare and Zhang [13] developed a security quiz and assessed risk tolerance in a gambling and lottery decision game. Using staff members in the business and finance department of a large public university, the authors reported that individuals who are intolerant of risk were more likely to regard legitimate contacts as phishing, but that participants who are more trusting (assessed via the portion of a $5 endowment they are willing to transfer to another participant who then has the option to transfer money back to them) and less curious (curiosity assessed by the willingness of a participant to pay for non-instrumental information in the game) perform better on a security quiz.

While this particular finding is promising for BIS theory development, methodological problems arise in the sending of actual phishing emails in studies and as part of a broader research agenda. First, Institutional Review Boards, who oversee and approve such research, may be uncomfortable with researchers deceiving participants into providing working usernames and passwords. The propensity for individuals to re-use log-in information across multiple accounts, coupled with the ubiquity of digital security, makes *any* active username/password combination an exceptionally sensitive piece of information. Moreover, ethical guidelines governing deception in human participant research specify the need to ‘debrief’ participants afterwards by explaining the deception, correcting participants’ misconceptions, and taking steps to minimize any adverse effects. It may not be feasible or appropriate to conduct such a dialogue remotely, via e-mail. Second, the phishing methodology limits the assessment of secure and insecure behavior to just one type of insecure action (i.e., clicking a link in a phishing email). Methodological advances may stem from the development of techniques that are viable in a broad number of BIS studies and provide measurements of several secure and insecure cyber behaviors.

One way to expand is through self-report. In self-report assessment, individuals are presented a list of statements/attributions (e.g., “I am neurotic”, “I don’t open spam email”, “I update my software regularly”) and asked to select a response option describing the degree to which each item applies to them or the extent to which they agree with the statement/attribution. For example, McBride et al. [1] reported on the likelihood of individuals’ intention to violate cybersecurity policies from personality traits, sanctions on non-compliance, and deterrence theory (i.e., likelihood of severe punishment). The probability of violating policy was determined by presenting participants with short written vignettes. Personality traits were associated with participant choices on the vignettes (e.g., individuals high in extroversion were more likely to violate, those high in conscientiousness or openness were less likely to violate).

Russell et al. [14] developed a self-report, the iSECURE, that drew from online security recommendations made by various university information technology departments, internet service providers, security software firms, and the United States Computer Emergency Readiness Team (US-CERT). The iSECURE focused on those recommendations intended for end users, rather than system administrators. These were crafted into reflective statements about user behavior (e.g., “I download security updates for my computer”, “I download general software updates”, “I would use software to access private/secure sites if there was a good reason”, “I would use software to access private/secure sites”, “I have downloaded malicious software by accident”), that could be rated in terms of personal applicability. A team of cybersecurity and behavioral science experts created and reviewed potential items in consultation with professionals in the field and pared the set to a final list of items. This final set includes statements describing both secure and insecure end user cybersecurity behaviors (i.e., the iSECURE assesses not only passive insecure behaviors, but also the tendency to engage in active behaviors that may form the basis for insider attack).

In the initial study of the iSECURE [14] results of factor analysis suggested two factors consistent with theory: one underlying secure behavior items and one underlying insecure behaviors. Distributions of these two scales suggested that while the secure behavior scale is positively skewed, such that individuals tend to report that they engage in secure practices (i.e., more in the high secure tail of the distribution), insecure behavior is normally distributed (with a few individuals possibly at extreme risk). Russell et al. further found that self-report of secure cyber behaviors was associated with lower levels of neuroticism. Conversely, insecure cyber behaviors were associated with lower levels of conscientiousness, higher levels of psychological symptoms such as somatic and depressive symptoms.

Self-report methodology allows assessment of a number of secure and insecure cyber-behaviors and is widely used by psychological and behavioral science researchers [15]. However, in BIS investigators have been more dubious about the utility of self-reports. For example, Crossler et al. [16] caution against the use of self-report to assess what might be considered unethical cyber behavior (e.g., willingness to engage in hacking), stating that, “people are not generally willing to admit committing [unethical] behaviors,” (p. 96). If, as described by Crossler et al., participants are unlikely to admit to engaging in unethical cyber behavior, the distribution of scores should show severe positive skew (i.e., the vast majority of respondents failing to endorse engaging in unethical behavior). Russell et al. [14], however, found that score distributions suggest relative willingness to report insecure behaviors and failure to engage in secure behavior. Notably, the study did not examine links to non-self-reported actual behaviors or decisions.

Indirect measures refer to actions or behaviors that are *associated* with the actual actions or behaviors in question but are *not* the actual behaviors themselves. Self-report can be used to measure indirectly, as in the Safe Computing Scales developed by Anderson and Agarwal [17], which measures concerns about cybersecurity with questions like, “How concerned are you that hackers might harm American corporations or the government by breaking into their computers?” and individual’s self-efficacy in engaging in secure cyber behaviors, “I feel comfortable taking measures to secure my primary home computer.” These items do not ask if one *engages* in the behaviors, but rather their feelings about the issue (cybersecurity) the behaviors target.

There are also a number of non-self-report indirect measures. For example, respiratory sinus arrhythmia is considered a physiological measure of emotion regulation and linked to mental health and personality [18]. Another such indirect measure is the latency of participant responses to stimuli presented in a dot-probe task, a common paradigm in psychological research. The task involves the simultaneous, rapid presentation (e.g., 500ms) of two stimuli with different emotional valence (e.g., two words or images—one neutral, the other threatening) on a screen (e.g., one on left, one on right, top or bottom). Immediately following the removal of the words or images from the screen, a visual probe (e.g. an “X”) is presented in the same location as one of the preceding stimuli. The participant responds by pressing a key to indicate the position of the probe (left or right). The theory behind this assessment technique is that the faster the probe is detected, the more likely the participant was attending to the stimulus that was located in the same position as the probe. Therefore, shorter probe detection latencies for one category of stimuli (e.g., threatening versus neutral images) over another indicate a selective attention bias towards the category with the shorter probe detection latency (generally, shorter latencies across all stimuli may simply indicate greater attention to the task). Individuals with anxiety, for example, have shorter probe detection latencies for mild threat stimuli (a word like “fear”) and related biases in memory for non-attention to stimuli [19]. Aggressive and callous-unemotional individuals have been shown to demonstrate shorter detection latencies behind images of pain, distress, and suffering than non-callous individuals [20,21].

Research using the modification of attentional biases has suggested causal linkages [22–26]. Additionally, similar stimuli have been shown to prime behavioral actions and memories [27–29]. An exciting possibility is therefore that the dot-probe response latencies may predict user responses in cyber security scenarios in a manner similar to personality traits, and then may be used to prime more appropriate responses. Research to test this theoretical link is needed.

### Current study

In this paper, we report a study designed to fill a gap in the literature by developing an observational measure of secure and insecure cyber behavior for broad use in BIS research via a computer program. The program collects both dot-probe task responses as well as Susceptibility and Resilience to Cyber Threat (SRCT) via an immersive scenario decision program. The SRCT assesses a number of secure and insecure behaviors (e.g., do individuals actually choose to update anti-virus software when prompted?; do they open phishing emails as propensity to insider attack?; do they engage in malicious hacking?) through the identification of observable actions alongside a distractor task. We also examine preliminary links between SRCT responding and attention biases (indirect measure) as well as links to personality traits and self-repotted secure and insecure behavior. Specifically, we report on (1) the incidence and distributions of computer task responses that assess secure and insecure behavior in an immersive task (SRCT, a non-self-report assessment), (2) provide psychometric analysis of the immersive scenario decision program (the SRCT) through factor analysis and internal consistency, (3) test the links between standard personality tests, secure and insecure behavior using self-report, dot-probe response latencies, and the SRCT.

This research was supported by a grant from the National Science Foundation (NSF; USA). We note this because in our NSF application (a priori before data were collected) we predicted: 1) data for psychometric analysis and refinement of the measure assessing susceptibility to cyber threat would be generated and we predict obtaining statistically analyzable distributions (i.e., there would be individual variation and the responses could be summed to form measures of secure and insecure behavior), 2) the items forming the SRCT scales would have acceptable internal consistency estimates (coefficient alpha of .7 or above), 3) that we would be able to predict 12–25% or more of the variance in the SRCT measure with dot-probe-assessed attention biases and standard personality inventories. Existing BIS research has predominantly focused on the Big Five personality traits of openness, conscientiousness, extraversion, agreeableness, and neuroticism [12,30]. We sought to expand this by examining other potential psychological predictors in addition to the Big Five (here we focus on mental health symptoms of anxiety, somatization, and depression). As noted above, Russell et al. [14] reported that increased reports of somatic and depressive symptoms were related to self-report of insecure behavior on the iSECURE.

In addition, we based an additional hypothesis on our work with the self-report (iSECURE). Specifically, we predicted a two-factor solution would fit the data well, with items loading onto secure and insecure behaviors. Finally, we explored links between the iSECURE and SRCT and similar self-reports of cyber behavior (i.e., Safe Computing Scales [17]) to examine convergent validity. Drawing from Crossler et al. [16], one would predict a weak association between the self-report and the SRCT. We also explored the nature of self-report and SRCT response and how discrepancies between SRCT and self-report may be related to the psychological traits/symptoms of the users.

## Material and methods

### Participants

Data were collected from a sample of 190 adults (76% female), between the ages of 18–61 (mean age = 26.12). Reported race/ethnicity as White not Hispanic (78.9%), Hispanic (6.3%), “mixed ethnicity/other” (5.8%), Asian (5.3%), and African-American or Black (3.2%). Total family income reported by participants was 50% reporting yearly income above $50,000, 30% reporting income between $20,000 and $50,000, and 20% reporting yearly income below $20,000. When asked about the highest level of education completed, 79% of participants reported completing at least some college, 19% reported completing a graduate degree, and 2% reported their highest level of education completed was high school. Participants most commonly reported having between 6 years and 15 or more years of experience using the Internet (98%) with only four participants (2%) reporting less than five years of Internet experience.

#### Participant recruitment procedures

Participants were recruited from a Midwestern U.S. university and surrounding community via flyers and online (social media) announcements. Participants were asked to come into a computer lab to complete a computer task for a study on “multi-tasking, math computation speed, and computer work habits”. All participants reviewed and signed informed consent and completed a reading comprehension exercise prior to beginning the computer task. Participants were not excluded based on their reading comprehension score, and no potential participants were excluded from this study (however, we do examine the role of reading comprehension on the findings below). We did not actively recruit individuals with cognitive impairments or those who were institutionalized. Participation was voluntary, and all participants were informed that participation or refusal to participate would not affect them in any way. Participants were each compensated with a $20 gift card for their time. The university Institutional Review Board reviewed study procedures and approved the study.

### Procedures

The experimental protocol consisted of five phases: 1. Recruitment for a study on “multi-tasking, math computation speed, and computer work habits” (the hypotheses were not readily apparent to participants), study description, informed consent and then reading comprehension assessment; 2. Dot-probe tasks (collecting response latency biases to threat and aggression/callousness via standardized protocols); 3. SRCT; 4. Self-report measures; 5. Debriefing. Reading comprehension was assessed with three short passages (on Opera, Dolphins and an Unsinkable Ship; https://www.grammarbank.com/short-reading-comprehension-passages.html) and three comprehension questions for each.

#### Computer task assessments

The computer program designed for this study consisted of two parts (and is available at https://github.com/ahmirf/Susceptibility-and-Resilience-to-Cyber-Threat-SRCT). In part one, response biases using word and picture-based dot-probe tasks were completed [21,25]. Note that the pictures are removed from the program available at this link due to the publishing agreement for the images (see http://csea.phhp.ufl.edu/media.html). The dot-probe tasks involve the simultaneous presentation of two stimuli (words or images) on a screen (e.g., one on left, one on right, top or bottom). Immediately following the removal of the words from the screen, a probe (e.g. dot, or “X” or other signal to respond) is presented in the same location as one of the preceding stimuli. We calculated the attention facilitation index for negative/distressful images and attention facilitation index for positive images [21]. We also calculated median response latency for trials with the probe behind the neutral word, median response latency for trials with the probe behind the threat word, and attentional bias to threat (Threat Bias Index) per MacLeod et al. [31].

The second part of the computer program assesses **Susceptibility and Resilience to Cyber Threat (SRCT).** SRCT was designed to measure susceptibility to cyber threat, propensity to insider attacks, and malicious hacking as well as responses that may protect against threat via direct behavioral responses in an immersive “game” environment. Susceptibility to cyber threat and propensity to insider attacks and malicious hacking is measured via responses to emails, interactions with security dialogs, and actions in a real-world simulation. For the purposes of the study, five character profiles were created with a name, date of birth (excluding year–the participants could choose the birth year), and address. Two characters were female, one was male, and one named “Pat” allowed users to select the gender for their character. These profiles were created so that participants could enter the character’s personal information instead of their own.

Participants were presented with this hypothetical situation for the immersive “game” environment:

Welcome to Smith Global Sales! As a valuable member of our accounting team, you’ll be helping us by determining how our competitors are actually doing relative to their published financial reports. To do this, we’ll be having you solve some accounting problems. We use software designed to track technology advances, product sales, and warehouse inventory at competing companies. Part of your new job is to monitor competitors’ stock prices and press releases so we can react to market changes in a timely manner. Of course, while doing so, you’ll also need to respond to emails and internal chat communications from co-workers.

The program simulates an accounting screen with simple math problems in the form of accounting sales and inventory report calculations. The math problems served as a “distractor” task and are based on established work by Hopko, et al. [32]. The participants’ responses from emails and prompts placed within the accounting screen simulation were the actual focus to determine cyber security tendencies. Participants are prompted to open emails, perform software updates, evaluate potential phishing schemes and malware, and also interact with other employees while performing ostensibly important (though simulated) math/accounting tasks wherein they are asked identify discrepancies in the reported inventory of competitor companies. For instance, one e-mail comes from a “new colleague,” and provides information about how to hack into a competitor’s system to gain more accurate information about the competitor’s inventory.

Some of the accounting tasks require the user to make a “guess” about missing data, which might be obtained by hacking other systems (thus, hacking may be to a user’s advantage). In a similar fashion, participants receive security prompts tasking them with downloading security software updates. Each of these “protective” activities carries a side effect of slowing down the user’s capacity to do the math tasks. As noted, some of the emails the participants receive allow them to illicitly access (i.e., hack) databases to retrieve information in order to complete the math problems. For example, one “hacking email” sent by a coworker, “Johnny,” provided the user with an improperly obtained username and password and encouraged the participant to use this information to inappropriately access a restricted database. Other messages resembled common phishing emails, inviting users to download a music player, get a free tour of a large company, or claim a monetary prize. Additional pop-ups included software updates, as well as a free iPad sign up window, which prompted users to share their email address. Finally, several pop-ups required users to enter their email address, passwords, gender, and date of birth.

For analysis, all items were coded 1 if the action constituted a theoretically insecure cyber behavior, and 0 if it did not. For example, in SRCT item one, “Windows”–Windows update needed, responses were coded 0 if the participant clicked to “update,” and 1 if the participant did not click on the link to update. In SRCT item six “Hacking1”–Hacking email sent by coworker, Johnny, the participant receives an email providing the participant with Johnny’s (illicitly obtained) username and password and encourages the participant to use this information to hack into a restricted database in order to complete the math/accounting problem on the page with missing information. “Hacking1” was coded 0 if the link was not clicked, and 1 if the link was clicked. Some items were combined when the presentation of an item depended on the participants’ actions in the initial item. For example, “Phishing3FORM” is only available if a participant clicks on the hyperlink in the “Phishing3” email. Thus, we combine variables that had dependent forms to create a three-level index. These combined variables then range from 0 (e.g., participant did not click on the link in the initial email and therefore never got the form), 1 (e.g., participant clicked on the link in the initial email, but chose not to fill out or submit the subsequent form), to 2 (e.g., participant clicked on the link in the initial email and filled out and submitted the subsequent form). The psychometric properties of this SRCT dependent variable are examined below in the results section to test the hypotheses noted above. Each action, its brief description, its related “item” and the percentage of individuals making the action are presented in Table 1. Note the order in the table is not the order of presentation in the program. The nature of the presentation can best be understood by reviewing the task program (as noted is available at https://github.com/ahmirf/Susceptibility-and-Resilience-to-Cyber-Threat-SRCT).

**Table 1:** Summary of SRCT potential secure and insecure actions, items on final scale and percentages completed by the sample (N = 190).

SRCT Item	SRCT Action	%
**1**	“Windows”–Windows update needed. Updated	38.4
**2**	“Adobe”–Adobe flash player update needed. Updated	26.3
***NA***	“iPad”–pop-up asking participants to enter their email address for a free iPad. Provided email	23.2
**3**	“Virus”–pop-up asking participants to run a virus detection assessment software. Healed	49.5
**4**	“WindowsNew”–Windows update needed by restarting your computer. Restarted	8.4
***NA***	“Java”–pop-up indicating that a new version is available asking participants to update their current version. Updated	97.4
**5**	“Phishing1”–Phishing email one looks like it comes from the company’s IT Department, telling participants there was a security breach detected and the user may no longer be able to send messages or files. There is a hyperlink for participants to reset their email password. Clicked hyperlink	65.3
**5**	“Phishing1FORM”–Form that appears when participants click on the link in the Phishing email one. Submitted old and new passwords.	48.4
**6**	“Hacking1”–Hacking email sent by coworker, Johnny. This email provides the participant with username and password and encourages the participant to use this information to hack into a restricted database. Clicked hyperlink	38.9
**6**	“Hacking1Form”–If this link is clicked in hacking email one, the participant is able to use provided username and password. Hacked to get information	35.8
**7**	“Phishing2”–Phishing email participant has won $20,000. A hyperlink in the email invites participants to click to receive their prize. Clicked hyperlink	5.8
**7**	“Phishing2FORM”–Invites participants to enter their character’s personal information to receive the $20,000 prize. Submitted information	4.7
**8**	“Defender”–Windows defender pop-up to scan for viruses. Completed Scan	63.5
	“Hacking2”–Hacking email sent by coworker, Joanne. This email again invites participants to click on a link in order to hack into a restricted database. If the link is clicked, there is no form to fill out, however, participants are given information missing from the accounting screen (math problems). Clicked hyperlink	65.1
**10**	“Email1”–This phishing email informs participants that they can win an Xbox 3. Clicked hyperlink.	4.3
**10**	“Email1FORM”–Participants who click on the Email1 hyperlink are taken to a form to enter their character’s personal information. Entered information	3.7
**11**	“Hacking3”–Hacking email two again appears to be sent by another coworker, Joanne. This email again invites participants to click on a link in order to hack into a restricted database. If the link is clicked, there is no form to fill out, however, participants are given information missing from the accounting screen (math problems).	74.3
**12**	“Email2”–is written in all capital letters and informs participants that system viruses are detected and they should click a hyperlink to reset their passwords. Clicked hyperlink.	19.9
**12**	“Email2FORM”–Participants are taken to this form when they click on the hyperlink from Email2. Submitted their password in the form.	10.8
**13**	“Phishing3”–This phishing email comes from “MarkZuck@facebooks.com” stating that “Facebooks, Inc.” is sponsoring a private tour of its company, and that the company that the participant is working for has contacted them and the participant was selected for the tour. If the participant wishes to participate in the Facebooks, Inc. tour, they are instructed to click the hyperlink in the email. Clicked link	53.8
**13**	“Phishing3FORM”–When a participant clicks on the hyperlink in the Phishing3 email, they are then asked to fill out a form with their personal information. Submitted information.	44.6
**14**	“Music”–Phishing email to download a free music player. Clicked hyperlink.	3.8
**14**	“MusicFORM”–Fill out this form in order to receive their free music player. Completed form.	3.2

*Table 1 Notes. NA* = Not included after Factor Analysis, Actions with the same item number were combined

After completing the computer tasks, participants completed the self-reported measures on the same computer using an online format.

#### Self-report measures

Descriptive statistics for self-reported variables of interest are presented in Table 1.

#### iSecure

As noted and described above, an 18-item instrument asks participants to rate the frequency with which they engage in behaviors along a four-point Likert-type scale (“Never”, “Rarely”, “Sometimes”, “Often”). Internal consistency of the 18-item scale in the current study was insecure behavior *α* = .87; secure behavior *α* = .76. Scoring on the iSECURE is such that higher scores on the secure scale represent more secure behaviors reported and higher scores on the insecure scale indicate more insecure behaviors reported.

#### Safe Computing Scales (SCS)

A measure developed by Anderson and Agarwal [17] was included as a means to assess convergent validity of the SRCT and iSECURE. In the following, we focus on the Security Behavior Self-Efficacy Scale which measures perceived ability to avoid cyber threat and the Cyber Security Concerns Scale which measures concerns about cyber threat. Higher scores represent more Security Behavior Self-Efficacy and greater Cyber Security Concerns.

#### Big Five Inventory (BFI)

The Big Five personality traits of openness, conscientiousness, extraversion, agreeableness, and neuroticism were assessed using the BFI [33,34]. Respondents are presented with 44 characteristics (e.g., “Is original, comes up with new ideas”) and asked to rate their personal applicability along a five-point scale (1 = “Disagree Strongly,” 5 = “Agree Strongly”). Subscale scores are created by averaging responses to items representative of each personality trait and higher scores represent greater levels of these traits. In the current study, internal consistency for each subscale ranged from *α* = .75 - .88.

#### Brief symptom inventory (BSI-18)

Participants were screened for symptoms of psychological disorder and distress using the BSI-18 [35], an 18-item self-report measure. The BSI-18 includes subscales assessing broad symptom dimensions of somatization, depression, and anxiety. The measure asks participants to rate how much that problem has “distressed or bothered” them in the past seven days on a five-point Likert scale (from “not at all” to “extremely.” Item examples include statements such as “faintness or dizziness,” “feeling tense or keyed up,” and “feeling blue.” Higher scores represent more anxiety, depression and somatization. In the current study, internal consistency for each subscale ranged from *α* = .82 - .85.

## Results and discussion

A complete, de-identified data file is available at: https://github.com/ahmirf/Susceptibility-and-Resilience-to-Cyber-Threat-SRCT. In order to develop final scoring for the SRCT, the factor structure of the SRCT was examined using exploratory factor analysis (principal axis factoring with Varimax rotation reported here). The goal was to determine the most theoretically consistent structure while minimizing cross loadings. The factorability of items appeared appropriate given the pattern of correlations among the items (i.e., many inter-item correlations exceeded .30) and the adequate sample size. Factor selection was guided by combined information provided by factor eigenvalues, variance predicted, and inspection of a scree plot. Loadings of .30 or greater were considered salient when reviewing factor solutions. Initially, a solution of 4 factors with eigenvalues above 1 emerged; however, the 4^th^ factor had only two items and there were significant cross loadings. Five, three and two factors were tested. A pattern emerged of secure and insecure items tending to load together, with two items failing to load saliently above .3 (i.e., the iPad phish) or to cross load (i.e., update Java). Factor analysis on the remaining 14 items (i.e., removing the iPad phish and update Java items) produced three factors with eigenvalues of 4.1, 2.2, and 1.7 with items loading on two insecure factors and one secure factor. Salient positive factor loadings are presented in Table 2. From this we created a 5-item secure actions scale and a 9-item insecure actions scale as viable measures to extract from the SRCT. Given the reverse scoring, higher scores on both do represent more insecure responding. We justified combining the two insecure action factors given that there was at least one salient cross loading between them, and because items did not appear to form theoretically distinct insecure behaviors (e.g., phishing and hacking items load on both scales).

**Table 2:** Salient positively loaded items from the factor analysis of 14 SRCT items.

	Factor		
	Insecure 1	Insecure 2	Secure
Windows Update			.780
Adobe Update			.718
Virus Alert			.313
Windows Update New			.442
Windows Defender			.501
Hacking Email 2	.798		
Hacking Email 3	.718		
COMphish1form	.547		
COMhack1form	.541		
COMphish2form		.759	
COMemail1form		.849	
COMemail2form	.381	.337	
COMphish3form	.622		
COMmusicform		.746	

*Table 2 Notes*. Action items from Table 1: Item 5 = COMphish1form, Item 6 = COMhack1form, Item 7 = COMphish2form, Item 10 = COMemail1form, Item 12 = COMemail2form, Item 13 = COMphish3form, Item 14 = COMmusicform.

**Fig 1:**
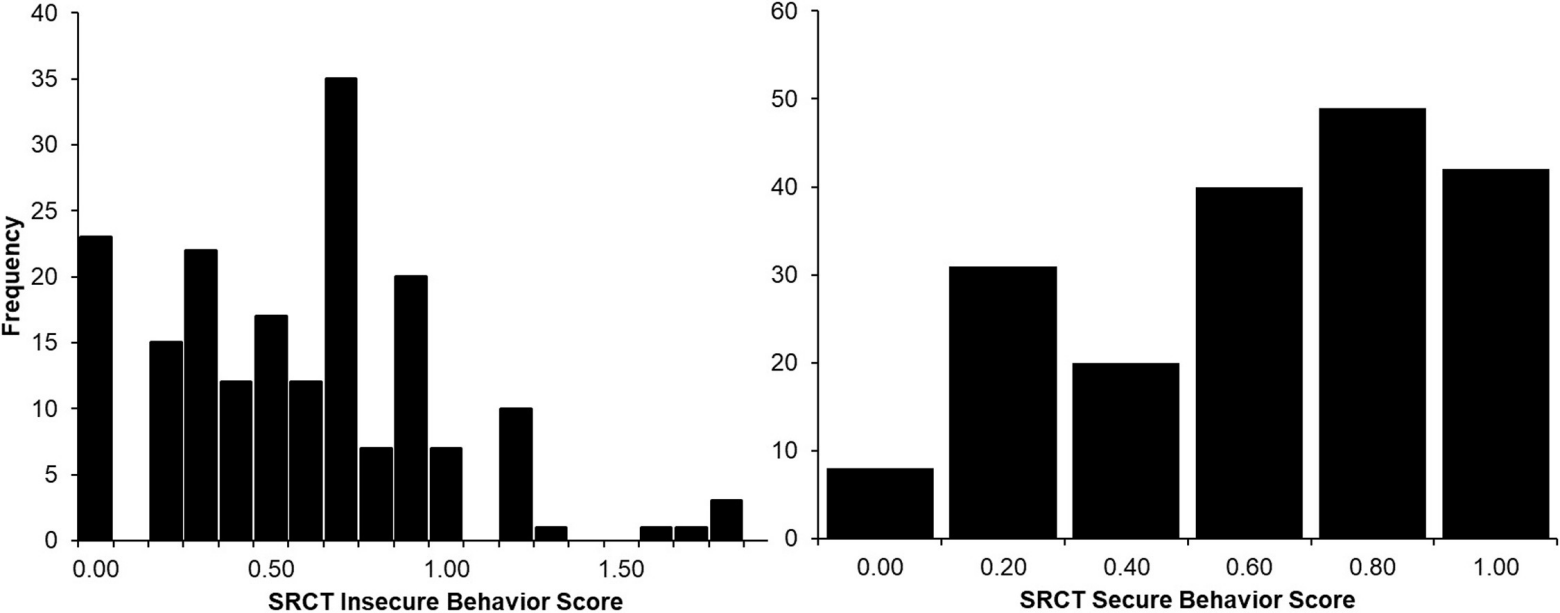
Distribution of the secure and insecure scales of the SRCT.

Scale scores were computed by taking the mean rating from each item, and in this way higher scores represent likelihood of endorsing the item. The mean SRCT secure scale score was .63 with a standard deviation of .30, while the mean SRCT insecure scale score was .53 with a standard deviation of .39. Distributions of these two scales are presented in Fig 1. Similar to the self-report data from Russell et al. [14], secure actions were common, however, insecure behaviors were less common–with four extreme outliers. Analyses using the SRCT insecure scale scores below are conducted both with the outliers and without these four cases. While the distributions are somewhat skewed, we did obtain statistically analyzable distributions (i.e., there is individual variation). As noted, we predicted acceptable internal consistency estimates (coefficient alpha of .7 or above). For the SRCT, internal consistency of the 5-item secure scale was .71 and the 9-item insecure scale was .76 (coefficient alpha). Age and gender were not associated with the SRCT secure scale score (age was not associated with the insecure scale as well). Males had higher mean scores than females on the SRCT insecure scale, but this appears to have been driven by the outliers because the difference was not significant when the 4 outliers were removed (3 of the 4 extreme cases were male).

**Fig 2:**
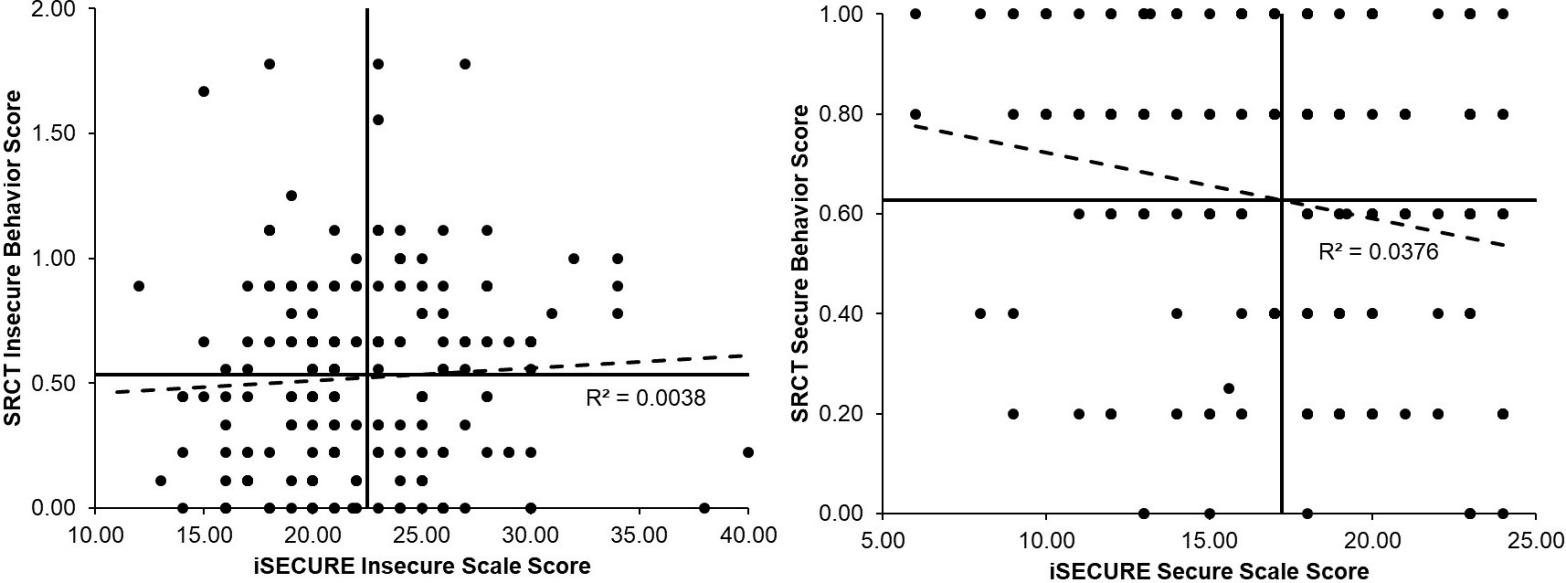
Scatter plots of iSECURE and SRCT scale scores with regression and mean score lines.

In terms of links between the SRCT scales and the link between SRCT scales and other variables, one salient finding is that the SRCT secure and SRCT insecure scales were significantly negatively correlated at *r* = -.40 (-.36 with outliers removed; both *p* < .01) suggesting there was a mild response bias toward taking actions regardless of the type of action. Correlations between the iSECURE and SRCT were not significant for the insecure scales (*r* = .05; .10 with outliers on the insecure scale removed) but the secure scales were significantly negatively correlated (*r* = -.19, *p* < .01), suggesting self-report of secure behaviors (where higher scores correspond to more secure behaviors reported) were related to secure actions in the SRCT (where higher scores correspond to *less* secure actions taken). However, this association was fairly weak. Scatter plots of iSECURE and SRCT scale scores with regression lines and mean scale score lines (i.e., the vertical and horizontal lines) are presented in Fig 2 and highlight the general patterns of scoring on the decision program versus the self-report.

The final a priori hypothesis was that we would be able to predict 12–25% or more of the variance in the SRCT measure with dot-probe assessed attention biases, standard personality inventories, and psychological inventories. Means and standard deviations for scores from self-report measures are presented in Table 3. Attention facilitation to positive images was very modestly (though significantly) correlated with SRCT secure responses (*r* = .15, *p* < .05). However, this may be driven by extreme scores on the attention facilitation index as a Spearman rank correlation (a correlational coefficient for ranked data and thereby addressing extreme scores) was not significant. The SRCT insecure scale was significantly negatively correlated with SCS cyber security concern scale scores at *r* = -.15 (-.16 with outliers removed; both *p* < .05), suggesting that those who made insecure responses in the SRCT were less likely to report cybersecurity concerns on the SCS. The iSECURE secure scale was significantly correlated with the Security Behavior Self-Efficacy Scale (*r* = .50, *p* < .001) and the Big Five Consciousness Scale (*r* = .16, *p* < .05). The iSECURE insecure scales were significantly correlated with the BSI Anxiety, Depression and Somatization Scales (*r* = .24, .21 and .19, respectively; all *p* < .01). We did not find additional patterns of significant correlations between the other self-report personality measures and SRCT or iSECURE. However, the pattern of associations among the other self-report measures was largely consistent with previous research and can be computed from the data file available online.

**Table 3:** Means, standard deviations, and ranges for the self report variables.

	Mean (SD)	Obtained Score Range
iSecure: Secure behavior	17.19 (4.43)	6–24
iSecure: Insecure behavior	22.48 (4.70)	12–40
Big Five: Openness	35.96 (5.87)	21–50
Big Five: Conscientiousness	34.12 (5.11)	19–44
Big Five: Extraversion	26.25 (6.80)	11–40
Big Five: Agreeableness	35.59 (4.96)	23–45
Big Five: Neuroticism	23.97 (6.00)	9–39
Trait anxiety	41.21 (10.07)	21–73
BSI: Somatization symptoms	2.67 (3.34)	0–19
BSI: Anxiety symptoms	3.99 (4.26)	0–20
BSI: Depression symptoms	3.93 (4.26)	0–20
Cybersecurity concern	33.05 (11.80)	8–56
Security behavior efficacy	21.56 (7.25)	5–35

We next looked at the effect of reading comprehension on the above results. We selected cases with 6 or more (9 total) correct answers on the test (*n* = 170). Overall, the pattern of factor structure and internal consistency remained highly similar. Internal consistency estimates of scores from the 5-item secure scale and the 9-item insecure scale were acceptable (*α* = .70, .73, respectively). A pattern emerged showing a similar set of associations. Again, the SRCT secure and SRCT insecure scales were significantly negatively correlated at *r* = -.34 (no difference with outliers removed; both *p* values < .01). Attention facilitation to both positive (*r* = .21, *p* < .01) and negative images (*r* = .19, *p* < .05) was modestly (but significantly) correlated with SRCT secure responses with the Spearman rank correlation approaching significance for positive images (i.e., controlling for outliers on the attention index, *rho* = .14, *p* = .079). Correlations between the iSECURE and SRCT scales were again not significant for the insecure scales but the secure scales were significantly negatively correlated (*r* = -.23, *p* < .01). Again, the SRCT insecure scale was significantly negatively correlated with SCS cyber security concern scale scores at *r* = -.19 (*p* < .05; no difference with outliers removed). The iSECURE secure scales were again significantly correlated with the Security Behavior Self-Efficacy Scale (*r* = .52, *p* < .001) and the Big Five Consciousness Scale (*r* = .18, *p* < .05). The iSECURE insecure scales were significantly correlated with the BSI Anxiety, Depression, and Somatization Scales (*r* = .27, .25 and .25, respectively; all *p* < .01).

### Exploratory analysis

**Fig 3:**
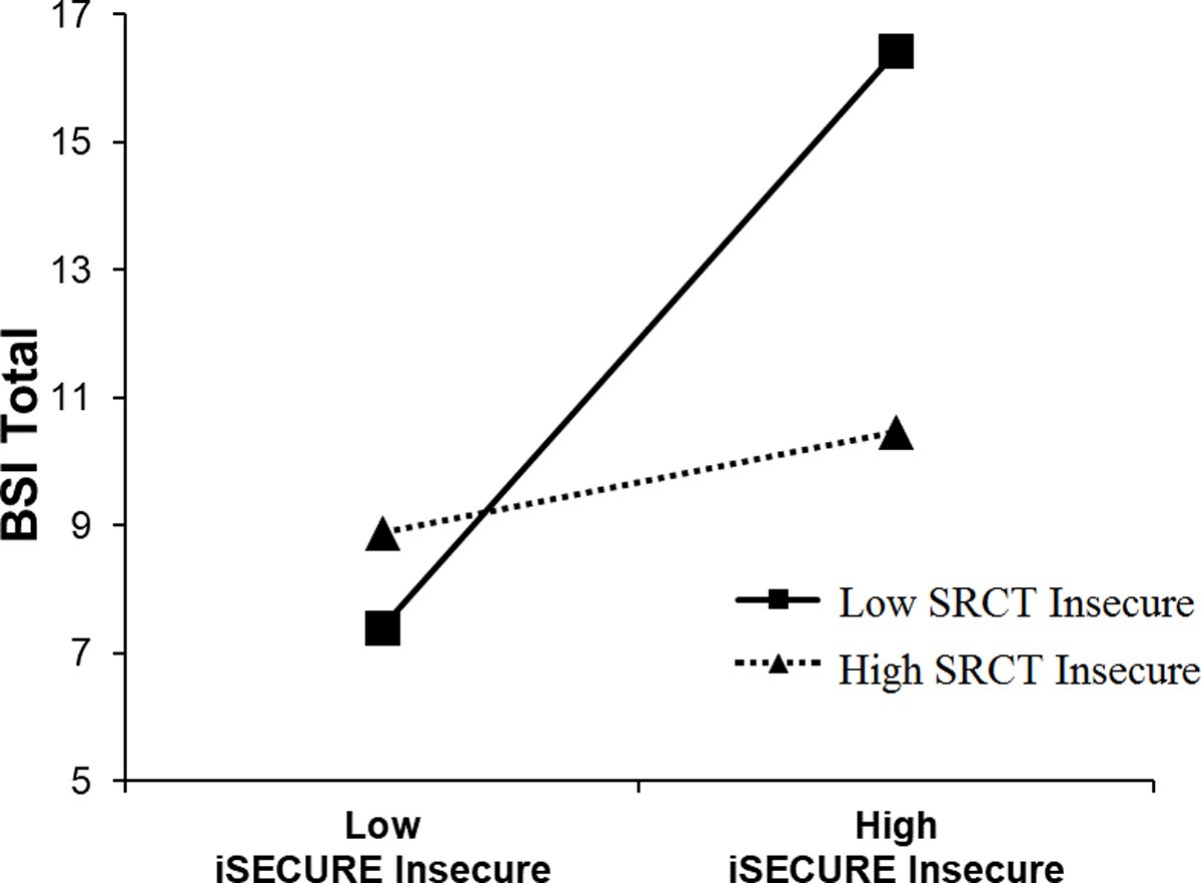
Association between iSECURE insecure scale scores and psychological symptoms as a function of SRCT insecure scores.

Given the relatively low correspondence between scores on the iSECURE self-report and SRCT scales (see Fig 2), we explored discrepancies between the self-report and actions in the SRCT were associated with BSI scores or attention biases. We used a series of regression analyses to formally test if discrepancies were predictive [36,37]. In each, we tested the corresponding SRCT scale and iSECURE self-report scale and an interaction term (e.g., iSECURE insecure by SRCT insecure) to predict. Predictors were centered and were entered simultaneously into the regression. Means and standard deviations for scores from self-report measures are presented in Table 3. One salient finding emerged as predictors of BSI scores (here we use the BSI total psychological symptoms for simplicity and recall above we found a positive association between insecure scale score on the iSECURE and BSI symptoms). In this analysis, the interaction term was significant (i.e., iSECURE insecure by SRCT insecure) and the nature of the interaction is depicted in Fig 3. Post-hoc testing (simple slopes analysis at plus and minus one standard deviation) indicated the association between iSECURE insecure and BSI psychological symptoms is positive and significant at low SRCT insecure actions (-1 SD; *β* = .43, *p* < .001), but not high levels (+ 1 SD; *β* = .08, *p* = .46). Interestingly, psychological symptoms were protective against SRCT insecure actions among those with high self-reported insecure behavior on the iSECURE. That is the SRCT insecure and BSI psychological symptoms are negatively associated and significant at high iSECURE insecure reports (+1 SD; *β* = -.29, *p* < .01), but not low levels (- 1 SD; *β* = .07, *p* = .48). We did not find interactions on the secure scales. This interaction finding was also present in the subsample with higher reading comprehension. While we did not identify other interactions, we would encourage additional exploratory analyses computed from the data file available online.

This paper adds to the literature by describing a novel technique to assess secure and insecure cyber behaviors, and provided initial data on this technique called the Susceptibility and Resilience to Cyber Threat (SRCT). The SRCT is an immersive scenario decision program to assess secure and insecure cyber-behavioral actions. The first hypothesis of obtaining statistically analyzable distributions (i.e., there is individual variation and the responses could be summed to form measures of secure and insecure behavior) was supported. Our second hypothesis was acceptable internal consistency estimates (coefficient alpha of .7 or above) and the SRCT scales both met this standard. The distributions of the two scales suggest that there is a skew towards people thinking they are engaging in secure cyber behavior (i.e., more in the high secure tail of the distribution) and that insecure behavior is also skewed toward people *not* making insecure decisions with possibly a few of very high risk (see Fig 1). A general tendency toward acting more secure is an interesting finding deserving more scrutiny. This was somewhat consistent with our previous self-report findings [14]. On a self-report measure people may actually be secure or they may have a bias toward feeling more secure than they really are. While the distributions of scores on SRCT decisions suggests actual decisions in a cyber-behavior scenario may partially comport to self-report general tendency toward secure actions/on-insecure, correlations between the iSECURE and SRCT scales were not significant for the insecure scales.

As noted, Crossler et al. [16] cautioned against the use of self-report to assess what might be considered unethical cyber behavior (e.g., willingness to engage in hacking). Russell et al. [14] found that in fact, people are generally willing to admit committing insecure cyber-behaviors on self-report and we also found willingness to engage in them in the SRCT. Indeed, 36% of the sample engaged in the first hacking opportunity and these numbers jump to 65% and 73% engaging in the second and third attempt, respectively (see Table 1). However, our correlation findings suggest that Crossler et al. [16] are justified to suggest wariness in self-report, as there were wide discrepancies in self-report and decisions in the SRCT. This was less true for the secure scales where we did observe convergent validity between the SRCT and iSECURE. Moreover, the SRCT insecure scale was very modestly associated with SCS self-reported concern over cyber security (lending some convergent validity to the SRCT and SCS). It may be that asking indirectly about general concerns about internet security may link to propensity to not engage in insecure behaviors more so than directly asking people if they engage in negative cyber actions.

The hypothesis that we would be able to predict 12–25% or more of the variance in the SRCT measure with dot-probe-assessed attention biases and standard personality inventories that assess personality traits (i.e., Big Five and psychological symptoms) was not well supported. Attention facilitation (more so in the subsample with higher reading comprehension) to both positive and negative images was very modestly correlated with SRCT secure responses with the Spearman rank correlation approaching significance for positive images. It may be that focused attention to the task at hand is associated with more secure actions (as opposed to response bias toward threat). Future research may wish to test this idea.

While links between personality traits and cyber behavior are emerging in the research literature [38–43], existing BIS research has predominantly focused on the Big Five personality traits of openness, conscientiousness, extraversion, agreeableness, and neuroticism. For example, Warkentin et al. [30] theorized that Big Five traits may interact with facets of Protection Motivation Theory (e.g., perceived sanction severity) to predict employee intentions to commit insider attack. Specifically, low conscientiousness paired with weaker beliefs about the severity and certainty of sanctions to predict greater likelihood that an employee might commit computer abuse. However, much of this research has solely relied on self-reported behavior, therefore the extant literature may overestimate the predictive ability of personality.

In this study, iSECURE secure scale was significantly correlated with the Security Behavior Self-Efficacy Scale [17] and the Big Five Conscientiousness Scale, while the iSECURE insecure scale was significantly correlated with BSI-reported psychological symptoms (similar to past research; [14,30]). However, our exploratory analyses suggest discrepancies between self-report and actions in the SRCT may be an interesting avenue to explore. Discrepancies were associated with psychological symptoms, with the association between iSECURE insecure and psychological symptoms positive and significant, but only at low SRCT insecure actions. In fact, results suggest that psychological symptoms may be protective against SRCT insecure actions but only among those with high self-reported insecure behavior on the iSECURE. Empirically, the reason for this is unclear from the study. Theoretically, one can speculate that there may be a general self-report response bias (i.e., multiple self-report measures are a common method–all self- report–and so the source variance leads to an artefactual association). There may also be a perception bias for general negative self-appraisals. That is, there is a well-established finding that depressed and anxious individuals tend to make general negative attributions about themselves, the world, etc.[44]. Such beliefs may generalize to individuals’ perceptions that they engage in negative cyber behavior, but this negative perception does not correspond to actual actions in the decision program. Future research should replicate the interaction findings and test these theoretical ideas before drawing firm conclusions.

### Limitations

Although the study contributes a new method and adds to the understanding of secure and insecure cyber behavior, there are limitations to the study. First, this study is limited by the cross-sectional nature of the investigation. Longitudinal research is needed to examine if SRCT scores are predictive of later secure or insecure actions in research similar to Halevi et al. [12]. That is, actual real-world decisions may not comport to actions in the decision program. Future studies can test this hypothesis, however, with the program. Second, drawing causal conclusions from the study is not warranted and so interpretation must be limited to associations (as opposed to causes). However, future experimental designs can be used to randomly assign participants to different conditions and use the program to test if experimental manipulations of conditions effect actions in the decision program. For example, do incentives of other conditions change probability of actions in the SRCT? Moreover, as noted above regarding the self-reports, because the study employed multiple self-report measures, there is the potential issue of source variance in reporting on these findings. Finally, the nature of the study may be improved by variation on the methods. For example, we did not use actual incentives to do well in the distractor accounting task game of identifying inventory discrepancies in the SRCT. Use of incentives may motivate more hacking, for example to get the information, or longer secure action times that interfere more directly with the putative objective of inventory accounting might identify those with propensity to true secure action (e.g. update software regularly).

### Conclusions

Overall, results from factor analysis, internal consistency, descriptive distributions and associations between SRCT scales were consistent with our theorizing and suggest the technique is viable as a construct measure or as an outcome variable in experimental intervention designs. The hypothesis that we would be able to predict 12–25% or more of the variance in the SRCT measure with dot probe assessed attention biases and standard personality inventories was not well supported but potentially important differences between self-reported and SRCT actions were identified. Indeed, past BIS research that has identified links between personality and cyber-behavior may be overestimating the links for actual actions. The program developed may be used in future research to expand tests of interventions for improving end user security and modified to answer additional related questions.
